# The Use of Virtual Reality as a Non-Pharmacological Approach for Pain Reduction During the Debridement and Dressing of Hard-to-Heal Wounds

**DOI:** 10.3390/jcm14124229

**Published:** 2025-06-13

**Authors:** Dariusz Bazaliński, Anna Wójcik, Kamila Pytlak, Julia Bryła, Ewa Kąkol, Dawid Majka, Julia Dzień

**Affiliations:** 1 Specialist Hospital, Podkarpacki Oncology Center of Fr. B. Markiewicz in Brzozów, 36-200 Brzozów, Poland; dbazalinski@ur.edu.pl (D.B.); kpytlak@ur.edu.pl (K.P.); 2 Institute of Nursing, Faculty of Health Sciences and Psychology, Collegium Medicum, University of Rzeszów, 35-959 Rzeszów, Poland; 3 Student Scientific Circle of Elderly Care, Institute of Nursing, Faculty of Health Sciences and Psychology, Collegium Medicum, University of Rzeszów, 35-959 Rzeszów, Poland; jb130749@stud.ur.edu.pl (J.B.); ek130885@stud.ur.edu.pl (E.K.); dm132766@stud.ur.edu.pl (D.M.); jd130806@stud.ur.edu.pl (J.D.)

**Keywords:** virtual reality, hard-to-heal wound, pain intensity, non-pharmacological methods

## Abstract

**Background/Objectives**: Pain management during minor surgical procedures in wound care across various etiologies is often underestimated in daily clinical practice. Pharmacotherapy remains the most effective and efficient method for pain reduction. However, growing concerns regarding the side effects of traditional analgesics and distressing psychosomatic experiences highlight the need for innovative non-pharmacological pain management strategies. The use of virtual reality (VR) has been suggested as a potential method to alleviate pain during medical procedures. The aim of this study was to assess the feasibility of virtual reality as a non-pharmacological approach to pain reduction during the debridement and dressing of hard-to-heal vascular wounds. **Methods**: This prospective observational study included a cohort of 100 patients who were consulted and treated at a specialized wound care clinic in the Podkarpacie region, Poland. Participant selection was based on predefined inclusion criteria. Patients were assigned to two groups: Group A, in which VR goggles were used, and Group B, in which standard care without VR was provided. All wounds were pre-treated with Lignocaine 2% gel for approximately 3–5 min before tissue debridement. Pain intensity was measured before the procedure, during the procedure, and 10 min after completion. A structured research questionnaire was used for data collection, consisting of two parts: sociodemographic data, functional assessment, wound characteristics, clinical scales, and the Numeric Rating Scale (NRS) for pain assessment before, during, and after the procedure. **Results**: A total of 100 participants voluntarily took part in the study, of whom 49.0% (n = 49) were male and 51.0% (n = 51) were female. The age of participants ranged from 43 to 89 years, with a mean age of 68.02 ± 10.0 years. A statistically significant difference in pain perception was observed between the pre-procedure and intra-procedure phases of wound debridement. The average pain increase in the Group with VR was lower than in the Group without VR (*p* = 0.006, effect size = 0.32). **Conclusion:** Pain occurrence and intensity during wound debridement are common challenges in clinical practice. The visual perception of a bleeding and treated wound may contribute to the psychogenic pain component. Virtual reality may serve as a simple adjunctive method to medical procedures by diverting attention away from surgical interventions. Further research, including psychological aspects of non-pharmacological pain management, is necessary in the context of wound care prevention and treatment.

## 1. Introduction

Chronic pain may affect up to 20% of patients worldwide, representing a widespread health concern and accounting for 15–20% of visits to healthcare facilities [1]. It is a complex and distressing phenomenon that is among the most common human experiences. According to the definition proposed in 2020 by experts from the International Association for the Study of Pain (IASP), pain is described as an unpleasant sensory and emotional experience associated with or resembling that associated with actual or potential tissue damage [2]. Pain in the course of chronic vascular injuries remains poorly understood, and treatment options and outcomes continue to be analyzed and refined in clinical guidelines and expert recommendations. Peripheral artery disease (PAD) is the most common vascular condition predisposing individuals to chronic pain with both somatic and neurogenic components. Persistent and debilitating pain can significantly impair health-related quality of life and contribute to existential suffering [3,4]. In diabetic foot disease, particularly in the presence of neuropathy, pain is relatively uncommon, as sensory disturbances often lead to callus formation, additional injuries, and secondary infections [5].

Pain associated with wounds can have various components (somatic, neurogenic, and psychological), resulting from tissue ischemia, nerve damage, inflammation induced by infection, as well as helplessness and limited ability to fulfill social and familial roles. Although pain is often discussed in the scientific literature, pain related to medical interventions within the wound area is rarely addressed or described. The pain component may be complex and arise from visual perceptions associated with the actions performed within the wound. Iatrogenic pain is associated with a lack of sensitivity, improper tools, or dressings used during the procedure. Pain can significantly slow down the wound healing process and decrease the patient’s quality of life [6]. Healthcare professionals should consider multimodal and multidisciplinary treatment protocols that address both physical and psychological aspects of care [3].

Although healthcare professionals are increasingly prioritizing patient pain management, the ability to effectively control pain remains insufficient. A holistic approach to treatment should be based on a safe and effective combination of psychoeducational methods and both local and systemic pain minimization strategies. It is unacceptable to ignore or inadequately document wound-related pain or to lack a structured strategy for monitoring and treatment aimed at improving the quality of life of individuals with chronic wounds. Pain management in chronic wounds depends on identifying the type and nature of pain, conducting a precise assessment, and systematically reporting and documenting the patient’s experience. The evaluation should be based on six key dimensions of pain experience: location, duration, intensity, quality, onset, and impact on daily activities [7]. The use of VR in clinical practice allows for immersive engagement in three-dimensional, dynamic environments, enhancing the focus on audiovisual stimuli. This distraction effect leads to improved pain tolerance during wound bed procedures. This technology may be beneficial for clinicians, as it has significant potential to improve psychological well-being [8]. An analysis of the literature reveals scientific evidence supporting the efficacy of virtual reality for pain relief in patients with wounds. In a meta-analysis of randomized controlled trials, Huang et al. demonstrated the efficacy of VR in young people, and those who used VR also showed lower levels of anxiety and less pain. The authors assessed pain in groups manifesting four types of pain, and the analysis showed lower levels of pain in patients who underwent the procedure using VR goggles [9]. The use of VR technology in current clinical practice is a point of interest for researchers worldwide, in patients with acute, procedural, and chronic pain.

## 2. Materials and Methods

### 2.1. Ethics

This study was approved by the Director of the Specialist Hospital at the Podkarpacki Oncology Center in Brzozów, Poland, and received a positive opinion from the Bioethics Committee at the University of Rzeszów (RESOLUTION No. 054/12/2024, dated 20 December 2024).

The study adhered to the guidelines of the Declaration of Helsinki. Participants were informed about the study’s purpose and provided written informed consent before participating. They were also given the option to withdraw at any time without providing a reason [8].

### 2.2. Subject

The aim of this study was to assess the feasibility of using virtual reality as a non-pharmacological method for pain reduction during the treatment and management of hard-to-heal wounds. The study design involved a prospective observation of patients conducted at a purposefully selected healthcare facility in the Podkarpackie Voivodeship (Wound Treatment Outpatient Clinic, Podkarpacki Oncology Center in Brzozów, Poland), which provides ambulatory care for patients with chronic wounds of various etiologies.

Patients with chronic wounds who provided informed consent after reviewing the study concept were enrolled in the study. The study included individuals with venous ulcers (classified as CEAP C6—active ulceration with a wound surface area ≥10 cm^2^) and patients with diabetic foot disease (DFD) who met the inclusion criteria (wound surface area ≥ 2 cm^2^, WIFI ≥ 1, due to the smaller wound size but a higher risk of deep tissue damage). Additional inclusion criteria were a minimum wound duration of four weeks but no longer than 18 months, a pain score on the Numeric Rating Scale (NRS) not exceeding 4 points, and no contraindications to participation (such as signs of systemic infection: fever > 38 °C, heart rate > 90 bpm, leukocytosis > 12,000 or <4000). Patients with hypoalgesia, pain exceeding 4 points on the NRS, or wounds of non-vascular etiology were excluded.

To determine the sample size necessary to detect differences, power analysis was performed with the following assumptions: effect size d = 0.6; alpha = 0.05; power = 0.8; two-tailed test. To calculate the sample size, the G*Power software was used. The desired sample size was 47 per group.

**Figure 1:**
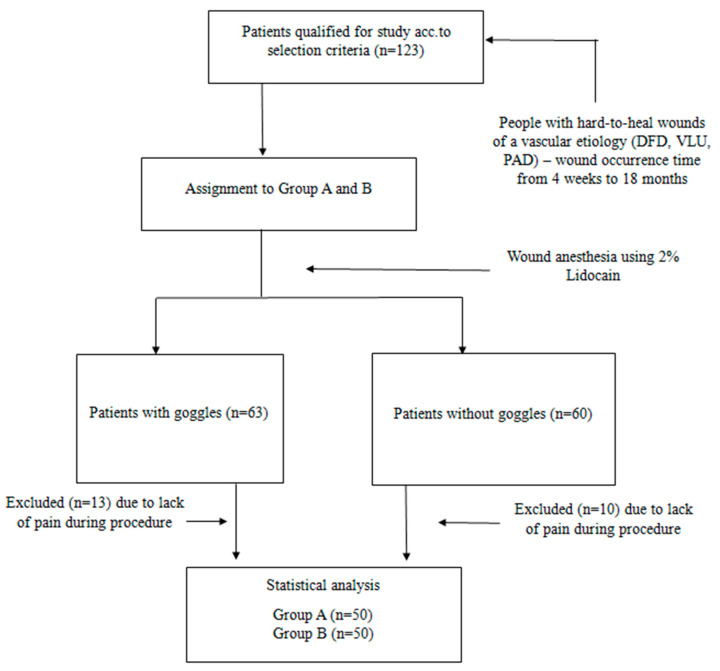
Process of qualification for the study.

Out of 365 first-time patients who were consulted and treated at the wound care clinic over a six-month period (2521 visits), individuals with neoplastic wounds, skin infections, pressure injuries, and complex postoperative wounds were excluded. Initially, 123 patients with diagnosed chronic injuries were enrolled in the study, of whom 23 were excluded, primarily due to a lack of pain during wound debridement (lack of pain sensation during scraping and/or necrotomy procedures). A pilot study was conducted from September to November (n = 25). The main study took place from December 2024 to March 2025. Participants from the pilot study were included in the final sample. The study ultimately involved 100 patients, who were assigned to one of two groups. Group A consisted of participants who used VR goggles, while Group B included those who did not. Group allocation was performed using the “random” function available in Excel 2019. Dressing changes were performed using an aseptic technique with surgical gloves, following the facility’s standard protocol. A 2% Lignocaine gel was applied to each wound for approximately 3–5 min before tissue debridement. Pain levels were measured before the procedure, during the procedure, and 10 min after completion. The study qualification protocol is illustrated in Figure 1.

### 2.3. Data Collection

The prepared scientific research questionnaire consisted of two parts. The first part included sociodemographic data (age, gender, place of residence, economic status, marital status, and education level). Additionally, the level of self-care was assessed using the Barthel scale. The next section evaluated the type of injury, its area, duration, and depth of tissue damage using a simple classification: partial-thickness, full-thickness, and deep tissue injury. As a supplementary tool, the (EPUAP/NPIAP *) scale was applied [10], while foot injuries were assessed according to the WIfI [11] and RYB classifications. The second part consisted of pain assessment tools, utilizing the Numeric Rating Scale (NRS) [12].

* The EPUAP/NPIAP classification was used due to the lack of a preferred wound assessment tool for lower leg injuries. This tool defines tissue damage as follows: 1°—superficial injury; 2°—partial-thickness skin injury; 3°—full-thickness skin injury; and 4°—injury with exposed tendon or bone.

**Figure 2:**
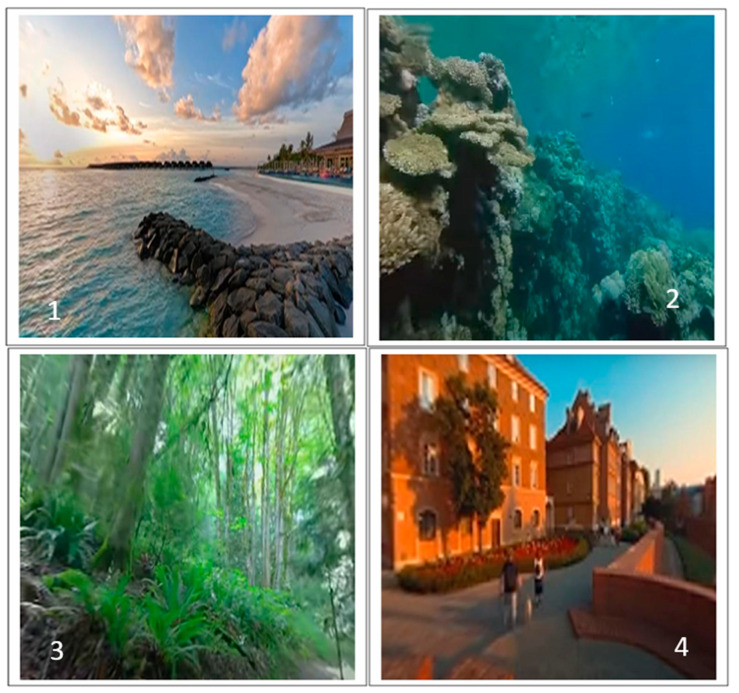
The most popular 3D images with sound available on websites, as selected by respondents: (**1**) https://youtu.be/jqq_ZdD5Zwg?si=7Ey51-96tgvwBxhx-VR (accessed on 9 June 2025); (**2**) https://youtu.be/eKumVFvGHFA?si=WMvX__jpwEUSiY1M-VR (accessed on 9 June 2025); (**3**) https://youtu.be/G_gmoSejUxU?si=nvT5i57mHCIb2kwB-VR (accessed on 9 June 2025); (**4**) https://youtu.be/FnpxXCRZ_Ps?si=EhuKFDFHQ_0qorsN-VR (accessed on 9 June 2025).

The study used Oculus Meta Quest^®^ 3 128 GB VR goggles (Meta Platforms, Inc., Menlo Park, CA, USA). To distract the patients, free short films available on YouTube were used, showing an exotic beach, a forest, the underwater world, and European cultural monuments (Figure 2). While watching the films, the patients did not perform any manual activities; they could look around, but they did not perform any activities that could affect the wound cleansing process. After discussing and completing the questionnaire, the nurse conducting the study collected information about the patient’s visualization preferences and then selected a film according to the patient’s choice. The film with sound was played a few minutes before the start of the wound cleansing. During wound treatment, the films were played in a loop if the cleansing procedure took longer than the film. During the study, hygienic masks were provided to patients who used Google VR, and the VR goggles were disinfected after each use with disinfectants designed for the equipment. Lidocaine 20 mg/g (2%) (30 g, Bausch Health Poland) gel was used for local anesthesia.

### 2.4. Statistical Analysis

Statistical analysis was performed using IBM SPSS Statistics v. 21. Descriptive statistics, histograms, box plots, and Kolmogorov–Smirnov tests for normality were used to assess the variables. Relationships between variables and verification of research hypotheses were analyzed using the following statistical methods: Spearman’s rank correlation (rho), Kruskal–Wallis tests, and Mann–Whitney U tests to examine differences in the distribution of dependent variables across independent variable categories.

## 3. Results

### 3.1. Characteristics of the Respondents

The statistical analysis included 100 participants, of whom 49.0% (n = 49) were men and 51.0% (n = 51) were women. Participants’ ages ranged from 43 to 89 years, with a mean age of 68.02 ± 10.0 years. The largest age group consisted of individuals aged 65–74 years (n = 45). Among those with chronic injuries, 38.0% (n = 38) lived in urban areas, while 62.0% (n = 62) resided in rural areas. The ability for self-care, assessed using the Barthel scale, had a mean score of 89.00 ± 12.53 points, with scores ranging from 40 to 100. According to established norms, 61.0% (n = 61) of participants had full self-care ability, while 39.0% (n = 39) exhibited deficits in this area. The data are presented in Table 1.

### 3.2. Wound Characteristics

In the study group, the most common wound types were those resulting from chronic venous insufficiency (VLU) (49.0%), mixed insufficiency (39.0%), arterial insufficiency (PAD) (6.0%), and diabetic foot disease (DFD) (10.0%). Injuries to the lower leg accounted for 86.0% (medial area; 55.0%, lateral area; 25.0%, circular area; 6%), with 14.0% affecting the foot. The average time since the wound’s onset, measured in months, was 7.16 ± 5.08, and the average wound area was 39.18 ± 71.83 cm^2^, with a minimum value of 2 cm^2^ and a maximum of 625 cm^2^. The analysis showed that 52.0% of the participants had full-thickness skin wounds, 40.0% had partial-thickness skin destruction, involving irregular epidermal and dermal damage, as a result of a wound area with irregular edges as well as excessive maceration and rupture of the tissues at the wounds, and 8.0% had wounds extending to tendons and bone. When assessed using the RYB classification, 67.0% of the wounds were red-yellow, and 22.0% were yellow. Detailed data are presented in Table 2. All participants underwent sharp debridement using basic surgical tools, such as scalpels, curettes, and scissors. Some 70.0% underwent scraping, 27.0% had scraping and removal of necrotic tissue, and 3.0% underwent necrosectomy.

### 3.3. Pain Management Treatment Provided

Upon analyzing the documentation and based on the interview collected from the patient, it was noted that 25.0% of the participants were taking painkillers regularly, with the first level of the analgesic ladder being most common (75.0%). Opioid analgesics were used by 23% of the participants, while strong opioids were used by 3.0%. The majority of the participants used over-the-counter (OTC) medications as needed for pain intensity. The data are presented in Table 3.

### 3.4. Pain Symptoms During the Wound Debridement Procedure

**Figure 3:**
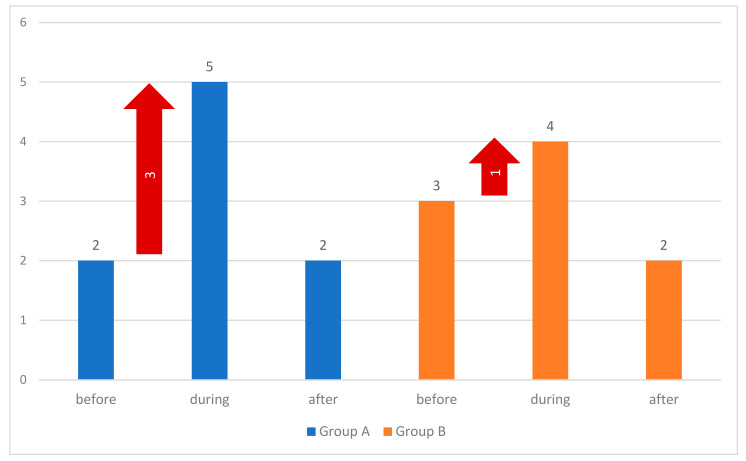
Median pain intensity in the NRS scale in Groups A and B, 10 min before wound debridement, during wound debridement, and 10 min after wound debridement. Red arrows indicate an increase in the median pain score on the NRS scale.

Pain sensations were assessed using the Numeric Rating Scale (NRS). The pain intensity did not exceed 4 points on the NRS before the therapeutic procedures, with a mean of 2.3 ± 1.8 in both groups. The assessment was conducted three times after the participants were familiarized with the evaluation principles. Statistical differences were noted in pain assessment before and during wound debridement (*p* < 0.05). In the goggle group (B), before the wound preparation, higher pain scores were reported than in the no goggles group (A). Ten minutes before the wound preparation, the average pain intensity in group B (VR goggles) was higher (2.60 ± 1.63) than in group A (2.0 ± 1.53). During the wound preparation, the pain intensity in group A was higher (4.94 ± 1.53) than in group B (4.32 ± 2.17). Ten minutes after the wound preparation, the pain intensity in both groups was similar: A (2.24 ± 1.41) and B (2.36 ± 1.71). The data are presented in Table 4 and graphically in Figure 3. The results allow for the hypothesis that the use of VR goggles reduces pain intensity (Table 5).

**Figure 4:**
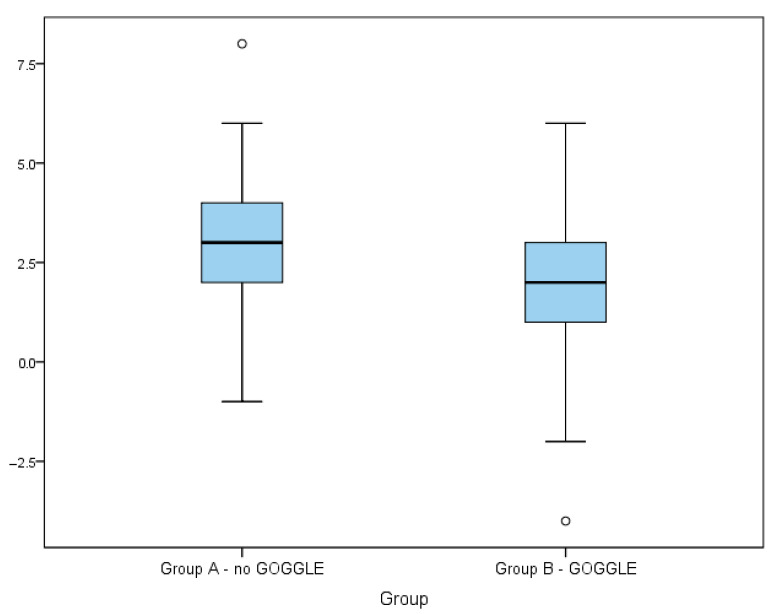
Box plot of the distribution of NRS value increase between measurements before and during wound debridement in Groups A and B.

As can be seen in Figure 3, the differences between pain levels before and during debridement are of particular interest. Descriptive statistics of distributions of NRS increase and the result of the test of the difference in the distributions of these increases in both groups are presented below in Table 5 and Table 6. In both groups, the mean values and the medians of the difference between NRS during debridement and before debridement are positive—in both groups, the average pain level increased during the debridement as compared to the values before debridement. However, the average increase in Group B is lower than in Group A. The difference between the mean pain value increase in both groups is 1.22 points (Table 7, Figure 4).

The Mann–Whitney U test was used to check the significance of the distribution differences between the groups (the distributions differ significantly from normal in both groups: Kolmogorov–Smirnov D(50) = 0.127, *p* = 0.042 for Group A and D(50) = 0.151, *p* = 0.006 for Group B). The test result showed an asymptotic significance (two-sided) at the level of *p* = 0.006, which indicates a statistically significant difference between the analyzed groups (*p* < 0.05) (Table 8).

### 3.5. Selected Variables and Pain Symptoms

In the case of pain assessment on the NRS scale, 10 min before wound debridement, no statistically significant differences were noted in pain assessment on the NRS scale between patients with different types of wounds, either in Group A or Group B (*p* > 0.05) (Table 9).

The relationship between pain assessment on the NRS scale, 10 min before wound debridement, and the time of wound occurrence and wound size were examined using the Spearman’s rho correlation coefficient. All correlation coefficients were low and statistically insignificant (*p* > 0.05). Therefore, there is no basis to claim that variables such as time of occurrence and wound size affect the pain level assessment 10 min before wound debridement (Table 10). No correlation was found between gender and pain intensity either; *p* > 0.05 (Table 11).

## 4. Discussion

Virtual reality has gained significant importance in recent years as an adjunctive method in managing pain of various etiologies, receiving positive feedback regarding its potential use in clinical practice. Its advantages in medicine and health sciences are evident, with positive outcomes in nursing education [13,14], skill acquisition [15,16], and clinical care [17,18,19], all supported by numerous studies. In the designed prospective observational study conducted within a wound care clinic, Oculus Meta Quest^®^ 3 goggles were used. The device was utilized in wound debridement and dressing procedures (scraping, necrotic tissue removal). From a group of 123 patients with chronic wounds of vascular etiology, the main exclusion criteria were hypo and hyperalgesia. A total of 100 individuals were qualified for analysis: 49.0% (n = 49) males and 51.0% (n = 51) females. The participants’ ages ranged from 43 to 89 years, with a mean of 68.02 ± 10.0 years. During the qualification process, patients were randomly assigned to two groups (A with VR goggles and B without goggles). Lignocaine gel was applied to the wound bed before the procedure for anesthesia. Most participants had wounds of venous and mixed etiology and were capable of self-care. Only 28.0% used painkillers regularly, while the rest used analgesics occasionally when the pain intensified. Pain intensity did not exceed four points on the NRS scale before the therapeutic procedures, with a mean of 2.3 ± 1.8 in both groups. In Group A (with VR), pain assessment before the wound treatment showed lower values (2.0 ± 1.53) compared to Group B (with goggles) (2.6 ± 1.63). During the procedure, Group B showed lower pain intensity (4.32 ± 2.17) than Group A (4.94 ± 1.53) (*p* < 0.05). In the final assessment 10 min after the procedure, a decrease in pain intensity was noted; Group A (without goggles) had a score of 2.24 ± 1.41, while Group B (with VR goggles) scored 2.36 ± 1.71. Although the difference in pain severity between the two groups is small, it may suggest clinical efficacy in alleviating procedural pain during chronic wound debridement procedures. In the VR group, the median NRS pain score increased by three points, while in the group without goggles, the median increased by one point. The results allow for the conclusion that using VR during wound debridement procedures reduces perceived pain intensity through distraction. No correlation was found between pain intensity and variables such as gender, type, area, or depth of tissue damage (*p* > 0.05). Our observations align with previous studies and clearly indicate the utility of VR in practice, supporting findings from other research [19,20,21,22]. During the wound debridement procedure, participants using VR goggles, particularly older individuals, showed significant interest in the images displayed, especially those depicting the sea and tropical island views with beaches.

The prevalence of chronic wounds correlates with lifestyle diseases and an aging population. These wounds are often associated with significant pain, which has a profound impact on quality of life. Studies indicate that the prevalence of chronic wounds ranges from 0.16% to 1.2% [21], with approximately 80% of individuals with chronic wounds reporting increased pain during dressing changes. The pain experienced by individuals with chronic wounds can be persistent, recurrent, and debilitating. It may occur even at rest, either continuously or intermittently, and often persists despite the use of commonly available analgesics. A meta-analysis conducted by Zhen-Hua et al. suggests that, in nursing practice, patients experience the most intense pain during dressing removal or changes, wound debridement, and the removal of necrotic tissue, compared to other nursing and therapeutic procedures. However, most studies analyzed in this review focused on acute burn wounds. A meta-analysis of 11 studies by Malloy and Milling found that VR distraction was effective in reducing experimental pain, as well as discomfort associated with burn care. Studies on needlestick pain provided less consistent results. The use of more advanced virtual reality technology, capable of fully immersing a person in a virtual environment, was associated with greater relief. Research suggests that VR distraction may be a useful tool for clinicians [23]. Pain perception in the management of chronic wounds is typically associated with inflammation, hyperalgesia, ischemia, or adherence of the dressing to the wound bed. While procedures related to wound treatment are secondary factors, they may also contribute to increased pain. A non-pharmacological intervention using VR technology has the potential to reduce pain intensity and perception during wound care; however, such an approach may extend the overall procedure time. Nevertheless, it contributes to improved patient comfort and satisfaction. This makes it a valuable solution that should be promoted and implemented in clinical wound care [20]. As a distraction therapy, VR technology engages patients’ perception through visual and auditory stimuli, thereby dispersing negative pain-related signals and their perception. Virtual reality is a potentially powerful tool for pain relief by enhancing psychological well-being, offering users a three-dimensional (3D) environment with multisensory stimulation. It can create therapeutic scenarios that are effectively utilized in clinical settings. By drawing the user’s attention into a computer-generated world, VR limits cognitive capacity for processing pain signals [23]. Engaging attentional resources in an immersive and interactive virtual environment can further regulate patient focus, reducing pain perception during dressing changes and leading to improved pain control outcomes.

Unfortunately, there is still no consensus on the mechanisms by which virtual reality alleviates pain responses. The hardware and software used in VR interventions vary across studies, and questions regarding the durability of effects and potential mechanisms of action require further investigation. De Araujo et al. suggest that a potential explanation for VR-induced analgesia may be the concept of a bidirectional ‘skin–brain’ communication axis, which is responsible for both the skin’s response to psychological stress (exacerbation of skin diseases) and negative psychological states (depression, anxiety) in response to chronic wounds that are difficult to heal [21]. Our observations and the results of the study suggest that the use of VR from the beginning to the end of the procedure helps patients remain calmer and more comfortable during wound cleansing and dressing. This is particularly important in the case of chronic wounds, which are often very painful and require regular dressing changes. As indicated in the literature, the use of virtual reality can reduce stress and anxiety, thereby improving the well-being of patients during medical procedures [24]. Numerous scientific studies clearly indicate that VR is a simple and effective non-pharmacological method of pain relief, and every effort should be made to implement it as a preventive tool in therapeutic procedures [4,25].

## 5. Conclusions

The occurrence and increased intensity of pain during wound debridement procedures are common phenomena in clinical practice. The visual perception of a bleeding and treated wound may contribute to the psychogenic component of pain. Utilizing virtual reality can serve as a simple adjunctive method in medical procedures, aiming to divert attention from the surgical process. The use of non-pharmacological methods to alleviate pain by diverting attention and focusing it on projected images can effectively improve the patient’s attitude towards the treatment process, as well as the procedures involved in wound debridement. Every effort should be made to continue further research, including psychological aspects, to explore non-pharmacological methods of pain reduction in the context of wound prevention and treatment.

### Limitations of the Study

1. Patients reporting NRS pain greater than four at the first assessment were excluded from the study due to the high risk of exacerbating pain during ongoing procedures
2. Patients who were taking opioid medication were excluded due to the risk of pain escalation as well as the risk of falsification.
3. During the study, 2% lidocaine gel was applied to the wound in both study groups for pain relief.
4. During the study, active distraction in the form of manual patient involvement was not used. During the study, in the group of patients who used Google VR, visualization with sound was used. Patients were allowed to look around during wound treatment if this did not interfere with the wound cleansing process.

## Figures and Tables

**Table 1:** Selected sociodemographic data in the study groups—gender, age, residence, and self-care capacity).

**Gender** ***p* = 0.317**	**Group**					
	**Total**		**Group A—No Goggles**		**Group B—Goggles**	
	**n**	**%**	**n**	**%**	**n**	**%**
Female	51	51.0%	23	46.0%	28	56.0%
Male	49	49.0%	27	54.0%	22	44.0%
Total	100	100.0%	50	100.0%	50	100.0%
**Age** ***p* = 0.075**	**Group**					
	**Total**		**Group A**		**Group B**	
	**n**	**%**	**n**	**%**	**n**	**%**
Up to 64 years	30	30.0%	19	38.0%	11	22.0%
65–74 years	45	45.0%	17	34.0%	28	56.0%
75+ years	25	25.0%	14	28.0%	11	22.0%
Total	100	100.0%	50	100.0%	50	100.0%
**Residence Location** ***p* = 0.039**	**Group**					
	**Total**		**Group A**		**Group B**	
	**n**	**%**	**n**	**%**	**n**	**%**
City	38	38.0%	24	48.0%	14	28.0%
Village	62	62.0%	26	52.0%	36	72.0%
Total	100	100.0%	50	100.0%	50	100.0%
**Self-Care Capacity** ***p* = 0.838**	**Group**					
	**Total**		**Group A**		**Group B**	
	**n**	**%**	**n**	**%**	**n**	**%**
I. 86–100—Self-care sufficient	61	61.0%	31	62.0%	30	60.0%
II. 21–85—Self-care deficit	39	39.0%	19	38.0%	20	40.0%
III. 0–20—Self-care insufficient	0	0.0%	0	0.0%	0	0.0%
Total	100	100.0%	50	100.0%	50	100.0%

**Table 2:** Wound characteristics: etiology, localization, and onset time in groups.

**Wound Etiology** ***p* = 0.189**	**Group**					
	**Total**		**Group A**		**Group B**	
	**n**	**%**	**n**	**%**	**n**	**%**
Venous leg ulcer (VLU)	49	49.0%	21	42.0%	28	56.0%
Mixed ulcer	35	35.0%	20	40.0%	15	30.0%
Peripheral artery disease (PAD)	6	6.0%	5	10.0%	1	2.0%
Ulcer secondary to diabetic foot disease (DFD)	10	10.0%	4	8.0%	6	12.0%
Total	100	100.0%	50	100.0%	50	100.0%
**Wound Localization** ***p* = 0.688**	**Group**					
	**Total**		**Group A**		**Group B**	
	**n**	**%**	**n**	**%**	**n**	**%**
Foot	14	14.0%	8	16.0%	6	12.0%
Lower leg (circumferential wound)	6	6.0%	2	4.0%	4	8.0%
Lower leg medial part	55	55.0%	29	58.0%	26	52.0%
Lower leg lateral part	25	25.0%	11	22.0%	14	28.0%
Total	100	100.0%	50	100.0%	50	100.0%
**Wound Onset Time** ***p* = 0.034**	**Group**					
	**Total (months)**		**Group A**		**Group B**	
Average	7.16		5.92		8.40	
SD	5.08		4.49		5.36	
Median	5.0		4.0		7.0	
Min	1.0		1.0		1.0	
Max	20.0		20.0		18.0	
Q1	3.0		3.0		3.0	
Q3	12.0		6.0		12.0	
n	100		50		50	

**Table 3:** Pain management treatment—types of medication according to WHO and painkiller use patterns.

**Types of Medication Acc. to WHO** ***p* = 0.358**	**Group**					
	**Total**		**Group A**		**Group B**	
	**n**	**%**	**n**	**%**	**n**	**%**
Level I	75	75.0%	38	76.0%	37	74.0%
Level II	23	23.0%	12	24.0%	11	22.0%
Level III	2	2.0%	0	0.0%	2	4.0%
Total	100	100.0%	50	100.0%	50	100.0%
**Painkiller Use Patterns** ***p* = 0.656**	**Group**					
	**Total**		**Group A**		**Group B**	
	**n**	**%**	**n**	**%**	**n**	**%**
Regular use	28	28.0%	13	26.0%	15	30.0%
As needed	72	72.0%	37	74.0%	35	70.0%
Total	100	100.0%	50	100.0%	50	100.0%

**Table 4:** Pain intensity measured on the NRS scale 10 min before, during, and 10 min after the debridement) procedure.

**10 min Before Wound Debridement**	**Group**		
	**Total**	**Group A**	**Group B**
Average	2.30	2.00	2.60
SD	1.60	1.53	1.63
Median	2	2	3
Min	0	0	0
Max	4	4	4
Q1	1	1	1
Q3	3	3	4
n	100	50	50
**During Wound Debridement**	**Group**		
	**Total**	**Group A**	**Group B**
Average	4.63	4.94	4.32
SD	1.89	1.53	2.17
Median	5	5	4
Min	0	2	0
Max	10	8	10
Q1	4	4	3
Q3	5	6	5
n	100	50	50
**10 min After Wound Debridement**	**Group**		
	**Total**	**Group A**	**Group B**
Average	2.30	2.24	2.36
SD	1.56	1.41	1.71
Median	2	2	2
Min	0	0	0
Max	9	5	9
Q1	1	1	1
Q3	3	3	3
n	100	50	50

**Table 5:** Normality tests of NRS scales distributions before, during, and after wound debridement in Group A and B.

Time of Measurement	Group	Kolmogorov–Smirnov		
		Statistic	df	Significance
10 min before	Group A—no goggles	0.240	50	0.000
	Group B—goggles	0.157	50	0.003
During	Group A—no goggles	0.204	50	0.000
	Group B—goggles	0.197	50	0.000
10 min after	Group A—no goggles	0.148	50	0.008
	Group B—goggles	0.194	50	0.000

**Table 6:** U Mann–Whitey test statistics—distribution comparison of NRS 10 min before, during, and 10 min after the wound debridement in Groups A and B.

	NRS		
	10 min Before	During	10 min After
Mann–Whitney U	945.000	968.000	1241.500
Wilcoxon W	2220.000	2243.000	2516.500
Z-score	−2.142	−1.985	−0.060
Asymptotic significance (two-sided)	0.032	0.047	0.952
Effect size (rank-biserial correlation)	0.244	0.226	0.007

**Table 7:** Descriptive statistics of the distribution of NRS value increase between measurements 10 min before and during wound debridement in Groups A and B.

NRS During—NRS Before Debridement	Group		
	Total	Group A	Group B
Average	2.33	2.94	1.72
SD	2.11	1.85	2.20
Confidence interval	1.91–2.75	2.42–3.46	1.09–2.35
Median	2	3	2
Min	−4	−1	−4
Max	8	8	6
Q1	1	2	1
Q3	4	4	3
n	100	50	50

**Table 8:** U Mann–Whitey test statistic—comparison of NRS increase 10 min before and during the wound debridement in Groups A and B.

Difference Between Groups A and B	Difference Between NRS During and Before Debridement
Mann–Whitney U	854.000
Wilcoxon W	2129.000
Z	−2.767
Asymptotic significance (two-sided)	0.006
Effect size (rank-biserial correlation)	0.32

**Table 9:** Descriptive statistics of pain assessment on the NRS scale 10 min before wound debridement and results of the Kruskal–Wallis test for distribution equality across wound types in Groups A and B.

NRS	Group							
	Group A—No Goggles				Group B—Goggles			
	Wound Type				Wound Type			
	Venous Leg Ulcer (VLU)	Mixed Ulcer	Peripheral Arterial Disease (PAD)	Other	Venous Leg Ulcer (VLU)	Mixed Ulcer	Peripheral Arterial Disease (PAD)	Other
Average	2.38	1.60	1.60	2.50	2.43	2.80	4.00	2.67
SD	1.77	1.05	1.14	2.38	1.77	1.42		1.63
Median	2	2	2	3	3	3	4	3
Min	0	0	0	0	0	0	4	1
Max	7	4	3	5	6	5	4	5
Q1	2	1	1	1	1	2	4	1
Q3	3	2	2	5	4	4	4	4
n	21	20	5	4	28	15	1	6
K-W test	H(3) = 2.482, *p* = 0.479				H(3) = 1.400, *p* = 0.705			

**Table 10:** Spearman’s rho correlation coefficients between pain assessment on the NRS scale 10 min before wound debridement and wound duration and extent in Groups A and B.

Rho Spearman	Group A		Group B	
Pain Assessment Acc. to the NRS Scale 10 min Before Wound Debridement	Wound Onset Time	Wound Extent in cm^2^	Wound Onset Time	Wound Extent in cm^2^
Correlation coefficient	0.009	−0.195	−0.106	−0.174
Significance (two-sided)	0.951	0.175	0.466	0.228
n	50	50	50	50

**Table 11:** Mann–Whitney U test result for equality of pain intensity distribution on the NRS scale in female and male groups.

	10 min Before	During	10 min After
Mann–Whitney U	1025.000	1233.000	1200.000
Wilcoxon’s W	2351.000	2458.000	2425.000
Z-score	−1.577	−0.116	−0.349
Asymptotic significance (two-sided)	0.115	0.908	0.727

## Data Availability

The original contributions presented in this study are included in the article. Further inquiries can be directed to the corresponding authors.
